# Fractionation mechanisms of rare earth element speciation in granitic weathering profiles: Metallogenic implications for the kuanyu ion-adsorption REE deposit, dechang, SW China

**DOI:** 10.1371/journal.pone.0329138

**Published:** 2025-07-29

**Authors:** Lijun Qian, Lihua Ou, Guoxin Li, Xuepeng Xiao, Bo Qian

**Affiliations:** 1 School of Civil and Hydraulic Engineering, Xichang University, Xichang, China; 2 Institute of Earth and Planetary Sciences, Chengdu University of Technology, Chengdu, China; Universidade de Lisboa Instituto Superior Tecnico, PORTUGAL

## Abstract

The Kuanyu ion-adsorption rare earth element (REE) deposit, Sichuan’s first economically viable resource of its type, remains underexplored in terms of REE fractionation and enrichment mechanisms within its weathering profiles. This study systematically resolves REE speciation patterns and light-to-heavy REE (LREE/HREE) differentiation processes, advancing the metallogenic framework fo r such deposits. Analytical results demonstrate: (1) A vertically progressive enrichment of LREE-dominated REE concentrations from bedrock to topsoil, with the fully weathered layer serving as the primary enrichment zone; (2) Weathering of primary REE-bearing minerals and subsequent secondary mineral formation as drivers of elemental redistribution; (3) Contrasting controls by clay minerals, iron-manganese oxides, and humic acids—clay minerals preferentially adsorb LREEs, while iron-manganese oxides exhibit stronger HREE affinity through inner-sphere complexation, and humic acids enhance HREE mobility via stable complex formation. These findings establish iron-manganese oxides and organic ligands as dual regulators of REE fractionation, refining predictive models for ion-adsorption REE exploration in granitic weathering systems.

## Introduction

Ion-adsorption rare earth element (REE) deposits, alternatively termed lateritic weathering crust-type REE resources, constitute a critical class of REE deposits characterized by their dominant supply of over 90% global heavy REEs, coupled with advantages including comprehensive elemental fractionation, extractability, and low mining costs [1–3]. Since their initial discovery in Jiangxi Province during the 1960s, these deposits have subsequently been identified across southern China, notably in Fujian, Guangdong, Hunan, Zhejiang, Guangxi, and Yunnan [4–12].The Dechang region, situated within the Mianning-Dechang REE metallogenic belt, exhibits exceptional potential for ion-adsorption REE mineralization [13–15]. Recent systematic investigations have revealed multiple deposits and occurrences in the Panzhihua-Xichang area, including the Kuanyu, Mayanshan, Mantoushan, Ayue, Shimacun, Madi, and Banchantian prospects [13–17]. These discoveries have catalyzed research on the roles of parent rock lithology in governing REE migration, enrichment, and fractionation processes within weathering profiles [13].

Theoretical frameworks for ion-adsorption REE deposits primarily address two domains: magmatic-hydrothermal influences on protolith evolution [18–20], and REE fractionation/migration patterns within weathering profiles [21]. Existing research emphasizes ion-exchangeable REE fractions [22,23], while other speciation modes—including water-soluble, Fe-Mn oxide-bound, organic-complexed, carbonate-associated, and residual phases [19–21]—remain underexplored regarding their roles in elemental differentiation. Notably, Fe-Mn oxides demonstrate critical adsorption capacities due to their extensive surface areas, exerting dominant control over REE enrichment and mobility in natural aqueous systems [24], as documented in the Renju deposit of Guangdong [25]. Emerging evidence suggests organic matter and carbonates may also modulate REE transport dynamics [26,27]. Current models attribute profile-scale REE fractionation primarily to ion-exchange processes, with secondary influences from Fe-Mn oxide interactions and organic complexation. Resolving the interplay between these speciation mechanisms proves essential for deciphering REE migration-enrichment pathways and optimizing extraction methodologies.

To resolve the LREE/HREE fractionation, migration, and enrichment dynamics within the Kuanyu weathering profiles, this study integrates systematic mineralogical and geochemical analyses of multi-phase REE speciation. The findings unveil previously unrecognized fractionation mechanisms governing REE redistribution, addressing critical knowledge gaps in lateritic weathering systems. By establishing correlations between REE speciation transitions and weathering intensity gradients, this work provides a refined metallogenic model to guide exploration and sustainable extraction of ion-adsorption REE resources in analogous subtropical terrains.

## Geological Background

**Fig 1:**
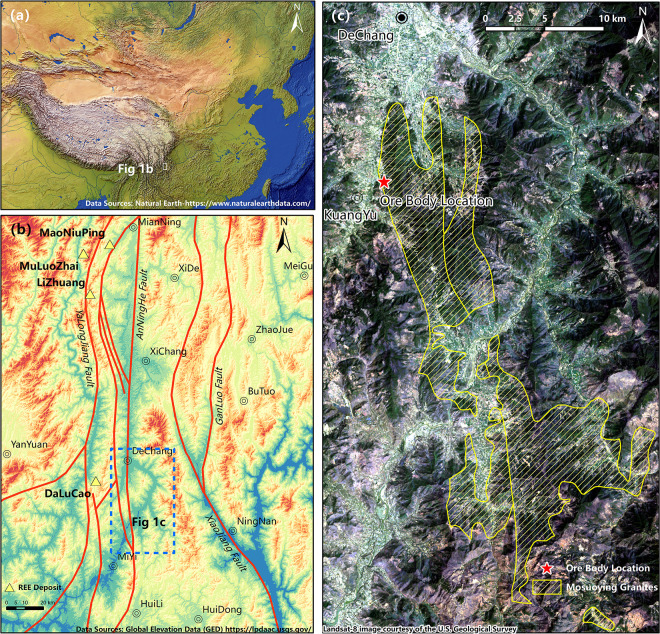
Regional Geological Map. a. Regional Location Map; b. Mianning–Dechang REE belt; c. Distribution of the Mosuoying granite and location of the weathering profile section.

The investigation area is situated in Dechang County, Sichuan Province, within the central Kangdian Axis along the western margin of the Yangtze Platform (Fig 1a). This region forms part of the Paleo-continental block bisected by the Anninghe Fault Zon, representing the core segment of the Mianning-Dechang rare earth element (REE) metallogenic belt e (Fig 1b). The Proterozoic metamorphic basement extensively outcrops, comprising Early-Middle Proterozoic lithostratigraphic units (Tangdan, Dahongshan, Hekou, Dongchuan, and Tong’an Groups) and Late Proterozoic sequences (Huili, Kunyang, and Yanbian Groups) (Fig 1c). Post-Proterozoic tectonic evolution involved intense magmatic episodes, including: Neoproterozoic (860–740 Ma) intermediate-acid magmatism and volcanic complexes [28–30]; Late Permian (260–259 Ma) mantle plume-related intracontinental rifting [31,32]; Middle Triassic (240–220 Ma) acidic-alkaline intrusions [33,34]. Himalayan alkaline magmatism (syenite-carbonatite assemblages) [35]. These multi-phase events shaped the Anninghe Magmatic Complex Belt along the NS-trending deep fault, hosting fluorocarbonatite veins with economic bastnäsite mineralization that defines the Mianning-Dechang REE belt. Notable deposits include the Maoniuping super-large, Dalucao large, as well as Muluo and Lizhuang medium-scale LREE resources [36–38] (Fig 1b). The belt’s granitoids exhibit elevated REE concentrations (average 370 ppm) [15], comparable to Jiangxi Province’s REE-enriched granites (Zhaibei 291–594 ppm [10]; Ningdu 144–272 ppm, average 198 ppm [39]; Shitouping 173–593 ppm [40]; Qingxi 201 ppm and Guanxi 175 ppm [41]; Zhangtiantang 95.25–180.94 ppm [42]) and exceeding South China’s regional average (229 ppm) [15].

The Kuanyu deposit derives its metallogenic material from the Mosuoying granite pluton (Fig 1c), emplaced during the early Neoproterozoic (842−790 Ma) [43]. This NS-striking intrusion extends 35 km in length with a ~ 10 km width, covering ~320 km² as the region’s largest composite batholith. Its multiphase emplacement history is dominated by coarse-grained biotite granite and porphyritic biotite granite variants [13], constituting the essential source reservoir for REE mobilization and subsequent ion-adsorption mineralization.

### Sampling and analytical methods

#### Profile characteristics and sampling strategy.

**Fig 2:**
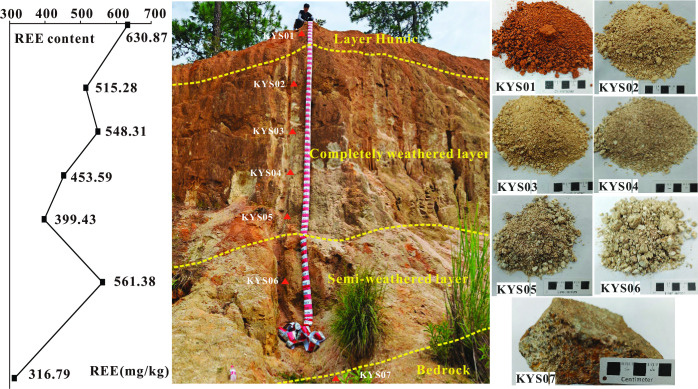
Schematic profile of the Mosuoying granite REE deposit and the variation of REE concentration along profile.

The well-preserved weathering profile at Kuanyu exhibits four vertically stratified horizons differentiated by color gradation and weathering intensity: bedrock, partially weathered zone, fully weathered zone, and humic topsoil (Fig 2).

Bedrock Horizon: Comprises relatively intact granitic masses undergoing initial physical disintegration.

Semi-Weathered Zone (2–3 m thickness): Gray to gray-white in coloration, this horizon retains discernible bedrock fragments within loosely consolidated matrices. Partial alteration of biotite and feldspars manifests as incipient kaolinization along mineral margins.

Completely Weathered Zone (6–10 m thickness): Displays progressive weathering intensification upward, transitioning from gray-white through yellowish-white to ochre and brick-red hues. Granite textures persist as relict structures in lower/middle sections, though reduced to friable sandy aggregates. Mineralogical transformations include: K-feldspar altering to pink clay minerals; Plagioclase converting to milky clay phases; Biotite degrading into flaky residual debris; Quartz existing as liberated granular particles.

Humic Topsoil (0.5–1.5 m thickness): Consists of ochre-colored soil rich in plant root systems, capping the weathering sequence.

In this study, systematic sampling was conducted across a weathering profile, yielding seven representative samples. Sampling site KYS01 was positioned in the surface humus layer at a depth of 0.2 m. Sites KYS02 through KYS05 were distributed at progressive depths within the fully weathered zone, specifically at 2.5 m, 4.1 m, 5.7 m, and 7.3 m, respectively. The deepest samples, KYS06 and KYS07, were collected from the semi-weathered zone and bedrock at depths of 9.6 m and 13.1 m, respectively. This stratigraphic sampling approach captures the vertical geochemical and mineralogical variations across the weathering profile.

### Analytical methods

Backscattered electron imaging (BSE) was utilized in this research to examine the mineral composition and microstructure of the specimens. The analysis was performed with a ZEISS SUPRA 55VP scanning electron microscope, which has an accelerating voltage range of 0.02–30 kV and a resolution of 1.0 nm at 15 kV. The microscope was equipped with a Backscattered Electron Detector (BSD) and supported a High Dynamic Range Backscattered Electron (HDBSE) imaging mode, enhancing the visibility of minerals with low atomic numbers. The samples were prepared as polished thin sections, approximately 30 µm thick, mounted on carbon conductive tape, and coated with a 10 nm layer of gold to prevent charge buildup. Imaging was carried out using backscattered electron signals, enabling the simultaneous acquisition of micro-area morphology through Secondary Electron (SE) imaging and elemental contrast via BSE imaging. To ensure the accuracy of the BSE images and the instrument’s stability, standard mineral samples (calcite and quartz) were analyzed for comparison, maintaining a grayscale fluctuation range within ±3% to ensure reliable mineral identification.

The analysis of major elements was conducted by Langfang Shangyi Rock and Mineral Testing Technology Service Co., Ltd., utilizing an ARL 9900XP X-ray Fluorescence Spectrometer (Equipment Number: YQ054). For method validation, geological reference materials (GBWs) were employed, including GBW07103 (silicate rock) and GBW07104 (granite). Each reference material was analyzed six times, with the deviations between the measured and certified values all within ±5%, indicative of the method’s high accuracy and reliability. The precision of the XRF analysis, assessed through six repeat measurements of the reference materials, demonstrated an RSD better than 5%. The results of the major element analysis are presented in Table 1.

**Table 1:** Major Element Composition of Weathering Profile Samples (in wt%).

Horizon	Layer Humic	Completely Weathered				Semi-Weathered	Bedrock
Sample	KYS01	KYS02	KYS03	KYS04	KYS05	KYS06	KYS07
K_2_O	3.99	5.79	6.02	5.83	5.49	5.06	4.392
Al_2_O_3_	20	15.84	13.12	13.07	12.71	11.94	11.9
CaO	0.059	0.044	0.049	0.07	0.082	0.468	0.424
TFe_2_O_3_	5.12	1.44	1.61	1.8	1.88	1.64	0.472
MgO	0.248	0.243	0.135	0.119	0.087	0.322	0.043
Na_2_O	0.206	0.123	0.19	0.383	0.537	2.52	3.795
SiO_2_	62.23	71.39	75.64	75.85	76.47	77.02	78.339
MnO	0.017	0.007	0.006	0.007	0.011	0.018	0.006
P_2_O_5_	0.055	0.042	0.039	0.032	0.028	0.04	0.006
TiO_2_	0.397	0.261	0.182	0.178	0.181	0.114	0.034
SUM	92.322	95.18	96.991	97.339	97.476	99.142	99.411
CIA	70.30	57.16	51.27	51.12	51.16	43.31	41.47

**Note**: CIA (Chemical Index of Alteration) CIA = 100 × [Al2O3/(Al2O3 + CaO + Na2O + K2O)]

wt% equals to 10^4^ mg/kg.

To investigate the speciation and enrichment patterns of rare earth elements (REEs) within the weathering profile, sequential extraction experiments were performed at the Testing Center of China Nonferrous Guilin Research Institute of Mining and Geology. These experiments isolated six distinct REE-bearing phases: water-soluble, ion-exchangeable, carbonate-bound, Fe-Mn oxide-bound, strong organic-bound, and residual fractions [44]. Inductively coupled plasma mass spectrometry (ICP-MS) was used to quantify REE concentrations in each extracted fraction, revealing depth-dependent variations in REE speciation and partitioning patterns (Tables 2 and 3).

**Table 2:** Rare Earth Element Composition of Weathering Profile Samples (in mg/kg).

Horizon	Layer Humic	Completely Weathered				Semi-Weathered	Bedrock
Sample	KYS01	KYS02	KYS03	KYS04	KYS05	KYS06	KYS07
La	108.42	109.08	101.55	90.06	78.64	83.12	58.58
Ce	247.71	150.94	232.2	161.66	121.56	272.14	112.23
Pr	26.27	25.26	22.11	19.78	17.62	18.55	13.18
Nd	94.12	89.28	80.16	73.01	65.26	68.39	49.23
Sm	18.11	16.42	14.23	13.49	12.46	13.18	9.48
Eu	1.55	1.43	1.24	1.14	1.08	1.07	0.82
Gd	18.01	15.74	14.09	13.35	12.30	13.8	9.45
Tb	2.77	2.46	2.10	2.08	1.99	2.157	1.58
Dy	14.20	12.66	10.14	10.14	9.87	10.86	7.76
Ho	2.94	2.70	2.12	2.11	2.08	2.305	1.70
Er	8.16	7.46	5.69	5.56	5.54	6.21	4.52
Tm	1.30	1.21	0.94	0.92	0.93	1.038	0.81
Yb	7.01	6.48	4.59	4.55	4.72	5.24	3.85
Lu	1.18	1.10	0.82	0.79	0.83	0.917	0.72
Y	79.135	73.0557	56.34	54.96	54.55	62.4	42.88
ΣREE	630.87	515.28	548.31	453.59	389.43	561.38	316.79
ΣLREE	496.17	392.41	451.48	359.14	296.62	456.45	243.52
ΣHREE	134.70	122.87	96.83	94.46	92.80	104.93	73.27
LREE/HREE	2.83	2.73	3.48	3.00	2.62	3.04	2.54
(La/Yb)_N_	10.14	11.04	14.51	12.98	10.93	10.40	9.98
(La/Sm)_N_	3.59	3.98	4.28	4.00	3.78	3.78	3.70
(Gd/Yb)_N_	2.11	2.00	2.52	2.41	2.14	2.17	2.02
δCe	1.08	0.67	1.14	0.89	0.76	1.61	0.94
δEu	0.26	0.27	0.27	0.26	0.27	0.24	0.26

**Note**: ΣREE = Σ(La—Lu); LREEs = La—Eu; HREEs = Gd—Lu; δCe = Ce/Ce* = CeN/(LaN × PrN)^1/2^, δEu = Eu/Eu* = Eu_N_/(Sm_N_ × Gd_N_)^1/2^, LREE/HREE = ΣLREE_N_/ΣHREE_N_, where subscript N represents normalization by Cl-chondrite [45].

**Table 3:** Distribution of Rare Earth Element Speciation Across Weathering Profile Samples (in mg/kg).

Speciation	Horizon Sample	Layer Humic	Completely Weathered				Semi-Weathered	Bedrock
		KYS01	KYS02	KYS03	KYS04	KYS05	KYS06	KYS07
Water-soluble REEs	La	0.013	0.0059	0.24	0.24	1.87	1.04	1.87
	Ce	0.036	0.0097	1.14	0.7	2.72	3.45	3.42
	Pr	0.0069	0.0033	0.055	0.058	0.43	0.23	0.42
	Nd	0.02	0.0057	0.19	0.2	1.57	0.85	1.52
	Sm	0.0064	0.0027	0.036	0.042	0.31	0.18	0.28
	Eu	0.0024	0.0022	0.0039	0.0043	0.021	0.014	0.014
	Gd	0.0058	0.0029	0.038	0.041	0.31	0.19	0.28
	Tb	0.0026	0.0023	0.0063	0.0073	0.046	0.03	0.036
	Dy	0.0055	0.0029	0.027	0.034	0.3	0.19	0.24
	Ho	0.003	0.0028	0.0065	0.0074	0.051	0.035	0.038
	Er	0.0038	0.0025	0.015	0.018	0.17	0.11	0.13
	Tm	0.0022	0.0021	0.0031	0.0035	0.018	0.014	0.013
	Yb	0.004	0.0028	0.013	0.016	0.15	0.1	0.11
	Lu	0.002	0.0019	0.0027	0.003	0.016	0.012	0.011
	Y	0.015	0.0057	0.13	0.16	1.75	1.18	1.47
	ΣREE	0.13	0.06	1.91	1.53	9.73	7.63	9.85
	LREE/HREE	0.78	0.43	3.79	2.81	2.39	2.47	3.10
	(La/Yb)_N_	2.13	1.38	12.11	9.84	8.18	6.82	11.15
	δCe	0.88	0.51	2.31	1.38	0.71	1.64	0.90
	δEu	1.20	2.40	0.32	0.32	0.21	0.23	0.15
Ion-exchangeable REEs	La	50.1	83.7	81.2	45.5	31	21.6	2.71
	Ce	48.3	35.4	57.9	8.31	2.47	2.92	0.51
	Pr	8.72	17.7	16.2	7.98	4.94	3.27	0.31
	Nd	29.3	61.8	58.5	29.6	18.6	12	1.01
	Sm	4.37	10.6	9.55	4.77	2.94	1.98	0.22
	Eu	0.36	0.7	0.57	0.36	0.27	0.22	0.086
	Gd	5	10.4	9.39	5.12	3.33	2.53	0.34
	Tb	0.68	1.43	1.2	0.72	0.49	0.39	0.12
	Dy	3.61	8.62	6.78	4	2.7	2.13	0.33
	Ho	0.75	1.58	1.18	0.76	0.55	0.48	0.15
	Er	2.17	4.99	3.74	2.23	1.6	1.37	0.28
	Tm	0.28	0.57	0.41	0.27	0.2	0.19	0.091
	Yb	1.48	4	2.72	1.55	1.03	0.9	0.2
	Lu	0.24	0.5	0.34	0.22	0.16	0.16	0.08
	Y	32	57.1	44.2	31.1	24.3	21.4	4.33
	ΣREE	187.36	299.09	293.88	142.49	94.58	71.54	10.77
	LREE/HREE	3.50	2.80	3.54	2.82	2.57	2.09	0.94
	(La/Yb)_N_	22.20	13.72	19.58	19.25	19.74	15.74	8.89
	δCe	0.54	0.21	0.37	0.10	0.05	0.08	0.13
	δEu	0.23	0.20	0.18	0.22	0.26	0.30	0.96
Carbonate-binding REEs	La	5.22	4.69	4.27	3.82	3.9	2.35	0.83
	Ce	6.16	2.83	5.13	2.09	1.02	1.06	0.55
	Pr	1.43	1.48	1.34	1.17	1.12	0.64	0.22
	Nd	5.2	5.7	5.36	4.51	4.41	2.41	0.64
	Sm	1.07	1.32	1.18	1.04	1.04	0.6	0.2
	Eu	0.14	0.16	0.14	0.13	0.14	0.12	0.083
	Gd	1.02	1.11	1	0.94	0.99	0.64	0.25
	Tb	0.19	0.22	0.19	0.19	0.2	0.15	0.1
	Dy	0.67	0.87	0.68	0.75	0.81	0.55	0.24
	Ho	0.2	0.23	0.19	0.2	0.22	0.18	0.13
	Er	0.39	0.52	0.39	0.41	0.47	0.36	0.2
	Tm	0.1	0.13	0.1	0.1	0.11	0.1	0.085
	Yb	0.3	0.49	0.35	0.34	0.37	0.28	0.17
	Lu	0.094	0.12	0.094	0.091	0.097	0.09	0.075
	Y	3.47	3.56	2.65	3.3	4.07	2.98	1.44
	ΣREE	25.65	23.43	23.06	19.08	18.97	12.51	5.21
	LREE/HREE	2.19	1.69	2.05	1.64	1.45	1.13	0.64
	(La/Yb)_N_	11.41	6.28	8.00	7.37	6.91	5.50	3.20
	δCe	0.52	0.25	0.50	0.23	0.11	0.20	0.30
	δEu	0.41	0.40	0.39	0.40	0.42	0.59	1.13
REEs associated with Fe-Mn (hydr)oxides	La	10.3	3.76	7.22	23	13.1	19.7	27.1
	Ce	93.3	53.8	103	97.1	60.3	126	57.9
	Pr	3.54	1.2	1.77	5.2	3.31	4.74	6.03
	Nd	13.2	4.34	6.33	19.3	11.7	17.5	22.7
	Sm	3.01	0.94	1.23	3.5	2.39	3.49	4.13
	Eu	0.28	0.13	0.14	0.22	0.19	0.25	0.21
	Gd	3.17	1.01	1.46	3.54	2.52	3.78	4.02
	Tb	0.49	0.19	0.21	0.47	0.38	0.56	0.55
	Dy	2.64	0.71	0.8	2.38	1.98	3.11	3.09
	Ho	0.53	0.21	0.2	0.44	0.39	0.59	0.53
	Er	1.45	0.43	0.42	1.15	1.08	1.71	1.63
	Tm	0.23	0.12	0.11	0.18	0.18	0.25	0.21
	Yb	1.23	0.42	0.39	1	1.02	1.54	1.38
	Lu	0.19	0.11	0.097	0.15	0.16	0.22	0.18
	Y	11.4	2.73	2.9	10.1	9.07	17.2	16.7
	ΣREE	144.96	70.10	126.28	167.73	107.77	200.64	146.36
	LREE/HREE	3.11	3.94	6.68	4.59	3.25	3.68	3.41
	(La/Yb)_N_	5.49	5.87	12.14	15.09	8.42	8.39	12.88
	δCe	3.60	5.90	6.71	2.07	2.13	3.03	1.05
	δEu	0.28	0.41	0.32	0.19	0.24	0.21	0.16
Humic acid-binding REEs	La	20.2	6.29	6.37	7.23	6.91	6.85	15.9
	Ce	45.1	20.6	51	22	17.6	20.9	30.2
	Pr	6.87	1.94	1.97	2.37	2.32	2.17	3.54
	Nd	25.9	6.99	7.24	8.45	8.47	8.01	13.5
	Sm	6.01	1.56	1.55	2.01	2.09	2	2.56
	Eu	0.48	0.17	0.16	0.19	0.2	0.21	0.18
	Gd	5.47	1.39	1.47	1.8	1.94	2	2.57
	Tb	0.9	0.26	0.25	0.34	0.37	0.38	0.39
	Dy	5.38	1.23	1.1	1.79	2.19	2.25	2.23
	Ho	1.01	0.3	0.25	0.36	0.43	0.47	0.43
	Er	3.16	0.75	0.62	0.99	1.3	1.39	1.3
	Tm	0.45	0.16	0.13	0.18	0.21	0.23	0.19
	Yb	3.17	0.8	0.62	1.07	1.39	1.45	1.12
	Lu	0.44	0.15	0.12	0.16	0.2	0.21	0.17
	Y	25.5	5.26	4.14	6.81	9.39	10.7	12
	ΣREE	150.04	47.85	76.99	55.75	55.01	59.22	86.28
	LREE/HREE	1.83	2.17	3.60	2.00	1.56	1.49	2.60
	(La/Yb)_N_	4.18	5.16	6.74	4.43	3.26	3.10	9.31
	δCe	0.89	1.37	3.35	1.24	1.02	1.26	0.94
	δEu	0.26	0.35	0.32	0.30	0.30	0.32	0.21
REEs associated with strong organic material	La	0.69	0.33	0.28	0.61	0.66	0.58	0.7
	Ce	3.01	1.8	2.83	2.96	1.85	2.81	1.05
	Pr	0.25	0.16	0.13	0.21	0.24	0.2	0.23
	Nd	0.7	0.34	0.26	0.55	0.61	0.52	0.6
	Sm	0.2	0.13	0.11	0.17	0.19	0.17	0.18
	Eu	0.086	0.088	0.084	0.083	0.091	0.086	0.078
	Gd	0.22	0.14	0.13	0.18	0.2	0.2	0.2
	Tb	0.096	0.093	0.087	0.093	0.1	0.097	0.1
	Dy	0.19	0.13	0.12	0.16	0.2	0.19	0.21
	Ho	0.11	0.11	0.1	0.11	0.12	0.12	0.12
	Er	0.14	0.11	0.096	0.11	0.14	0.14	0.15
	Tm	0.083	0.085	0.076	0.075	0.082	0.084	0.083
	Yb	0.14	0.12	0.1	0.12	0.15	0.15	0.15
	Lu	0.074	0.076	0.068	0.066	0.074	0.075	0.074
	Y	0.72	0.31	0.23	0.44	0.59	0.65	0.67
	ΣREE	6.71	4.02	4.70	5.94	5.30	6.07	4.60
	LREE/HREE	0.98	0.66	0.78	0.98	0.82	0.84	0.71
	(La/Yb)_N_	3.23	1.80	1.84	3.33	2.89	2.54	3.06
	δCe	1.69	1.82	3.45	1.92	1.08	1.92	0.61
	δEu	1.25	1.99	2.14	1.45	1.42	1.42	1.25
Insoluble REEs	La	21.9	10.3	1.97	9.66	21.2	31	9.47
	Ce	51.8	36.5	11.2	28.5	35.6	115	18.6
	Pr	5.45	2.78	0.64	2.79	5.26	7.3	2.43
	Nd	19.8	10.1	2.28	10.4	19.9	27.1	9.26
	Sm	3.44	1.87	0.57	1.96	3.5	4.76	1.91
	Eu	0.2	0.18	0.14	0.15	0.17	0.17	0.17
	Gd	3.12	1.69	0.6	1.73	3.01	4.46	1.79
	Tb	0.41	0.26	0.16	0.26	0.4	0.55	0.28
	Dy	1.7	1.1	0.63	1.03	1.69	2.44	1.42
	Ho	0.34	0.27	0.19	0.23	0.32	0.43	0.3
	Er	0.85	0.66	0.41	0.65	0.78	1.13	0.83
	Tm	0.15	0.14	0.11	0.11	0.13	0.17	0.14
	Yb	0.69	0.65	0.4	0.45	0.61	0.82	0.72
	Lu	0.14	0.14	0.1	0.1	0.12	0.15	0.13
	Y	6.03	4.09	2.09	3.05	5.38	8.29	6.27
	ΣREE	116.02	70.73	21.49	61.07	98.07	203.77	53.72
	LREE/HREE	4.63	3.57	1.56	3.71	4.45	5.64	2.50
	(La/Yb)_N_	20.82	10.39	3.23	14.08	22.79	24.80	8.63
	δCe	1.10	1.59	2.32	1.28	0.78	1.78	0.90
	δEu	0.19	0.31	0.73	0.25	0.16	0.11	0.28

**Note:** ΣREE = Σ(La—Lu); LREEs = La—Eu; HREEs = Gd—Lu; δCe = Ce/Ce* = Ce_N_/(La_N_ × Pr_N_)^1/2^, δEu = Eu/Eu* = Eu_N_/(Sm_N_ × Gd_N_)^1/2^, LREE/HREE = ΣLREE_N_/ΣHREE_N_, where subscript N represents normalization by Cl-chondrite [46].

This study employs a sequential extraction method to analyze seven chemical forms of rare earth elements (REEs) in ionic-type rare earth ores. The extraction protocols for each fraction are described below:

1. Extraction of water-soluble state (F1): Add 10 mL of water and oscillate for 3 hours at 25°C. After centrifugation at 20 minutes, separate the supernatant for analysis.
2. Extraction of ion-exchange state (F2): To the residue from F1, add 10 mL of water and 10 mL of 1 mol/L magnesium chloride solution (pH ≈ 7), then oscillate for 2 hours at 25°C. centrifugation for 20 minutes and separate the supernatant for analysis.
3. Extraction of carbonate-bound state (F3): Wash the residue from F2 with water, add 10 mL of 1 mol/L sodium acetate solution (pH ≈ 5), and oscillate for 5 hours at 25°C. centrifugation for 20 minutes to collect the supernatant for analysis.
4. Extraction of humic acid-bound state (F4): Wash the residue from F3 with water, add 20 mL of 0.1 mol/L sodium pyrophosphate solution (pH ≈ 10), and oscillate for 3 hours at 25°C. After centrifugation for 20 minutes, the supernatant is taken for analysis.
5. Extraction of iron-manganese oxide-bound state (F5): To the residue from F4, after washing with water, add 20 mL of hydroxylamine hydrochloride-hydrochloric acid mixture (0.25 mol/L hydroxylamine hydrochloride-0.25 mol/L hydrochloric acid), oscillate for 6 hours at 25°C. Centrifuge for 20 minutes and take the supernatant for analysis.
6. Extraction of strong organic matter-bound state (F6): Wash the residue from F5 with water, add 1.2 mL of 0.02 mol/L nitric acid solution and 2 mL of 30% hydrogen peroxide solution, and oscillate for 1.5 hours in a water bath at 83 ± 2°C. Then supplement with 1.2 mL of 30% hydrogen peroxide solution and continue oscillating for 1 hour at 83°C. After cooling to room temperature, add 2 mL of 1.6 mol/L ammonium acetate-1.6 mol/L nitric acid mixture, dilute the sample to 10 mL, and let it stand for 10 hours in a 25 ± 3°C environment. After centrifugation for 20 minutes, separate the supernatant for analysis.
7. Extraction of residual state (F7): Wash the residue from F6 with water, add 20 mL of nitric acid, 2 mL of sulfuric acid, 1 mL of perchloric acid, and 10 mL of hydrofluoric acid, and process according to the procedure for total analysis of ion-absorbed rare earth ore samples. Dilute to 100 mL with water and analyze the F7 extract.

After testing all samples, we statistically analyzed the recovery rates and obtained the range of 95.7–98.6%. The recovery Rate was calculated using the following formula 1:


Recovery\ Rate=∑F/Total×100% (1)

Total represents the total amount of rare earth elements (REEs) determined after complete digestion of the samples.∑F indicates the sum of the contents of the seven sequentially extracted fractions (F1–F7). Indicating that the sequential extraction method used is suitable for the chemical speciation analysis of REEs in ion-type REE ores.

For this determination of rare earth element analysis, the Thermo Scientific™ iCAP™ Q ICP-MS instrument was used without employing reaction gas. The same sample was divided into five equal parts, and five parallel determinations were performed. The determination of REEs used the Chinese National First-grade Reference Material GBW07158 as the quality control sample. The measured values were basically consistent with the certified values, and the results are shown in Table 4.

**Table 4:** Measured values and certified values of the standard mineral GBW07158.

Element	Y	La	Ce	Pr	Nd	Sm	Eu	Gd	Tb	Dy	Ho	Er	Tm	Yb	Lu	∑REE
Certified Value	142	264	74.9	40.7	146	28.6	7.00	27.5	4.59	23.8	4.98	14.0	2.10	12.4	1.76	794
Measured Value	140	261	74.2	41.1	143	28.6	7.11	28.1	4.63	23.2	5.08	14.4	2.06	12.1	1.82	786

**Note**: ΣREE = Σ(La—Lu).

The determination of REEs typically employs dual internal standards, ^103^Rh and ^185^Re, for calibration. The concentration of rhenium and rhodium in the experimental extraction solution is found to be very low, rendering their impact on the REE determination negligible. To evaluate the calibration effect of these internal standards, a matrix-matched mixed standard solution at 10.0 μg/L was prepared for the study. Concurrently, a mixed internal standard solution containing 5.00 μg/L each of ^103^Rh and ^185^Re was selected for the calibration procedure. The results demonstrate that when using the ^103^Rh-^185^Re dual internal standard calibration, ^103^Rh was used to correct for Y, La, Ce, Pr, Nd, Sm, Eu, and Gd, while ^185^Re was used for Tb, Dy, Ho, Er, Tm, Yb, and Lu. The recovery rates for these 15 rare earth elements ranged from 96.0% to 102%. In contrast, without the ^103^Rh-^185^Re dual internal standard calibration, the recovery rates for the same 15 elements were only between 90.0% and 97.0%. This indicates that the mixed internal standard solution effectively compensated for and corrected matrix effects and instrument drift encountered during the determination process.

## Results

### Mineral composition

**Fig 3:**
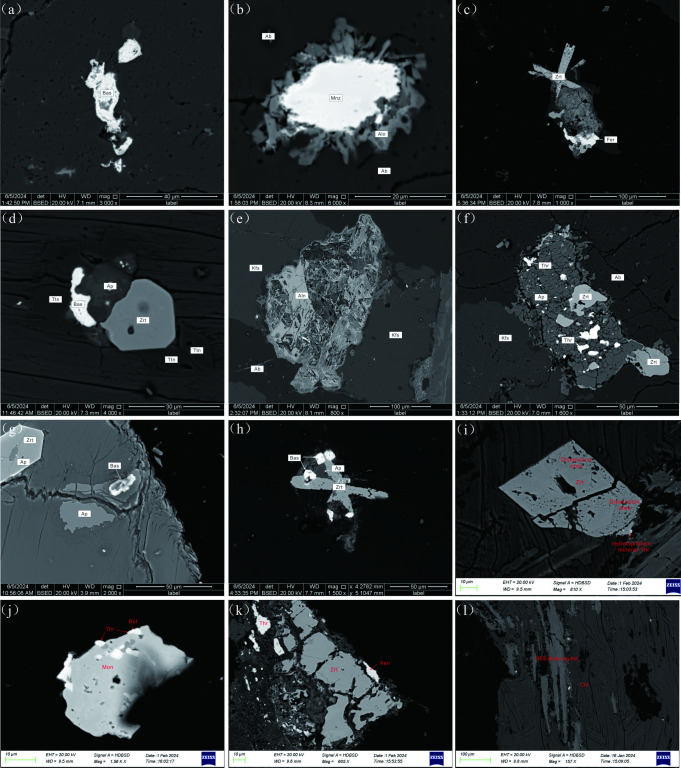
Backscattered Electron (BSE) Microscopy of Rock and Mineral Features. (a–h) BSE images of the bedrock: (a) Anhedral bastnäsite; (b) Monazite and allanite mineral assemblages, with allanite developed along the margins of monazite; (c) Zircon and ferrocolumbite mineral assemblages; (d) Zircon, apatite, sphene, and bastnäsite mineral assemblages. (e) Secondary alteration of allanite; (f) Apatite and thorite mineral assemblages, with apatite and zircon showing fragmentation and secondary thorite aggregates developed within apatite cavities; (g) Bastnäsite grown within apatite, with some apatite grains included as inclusions within zircon; (h) Zircon, apatite, and bastnäsite mineral assemblages; (i–l) BSE images of the semi-weathered zone: (i) Zircon showing fragmentation and dissolution, with secondary thorite aggregates developed along zircon margins; (j) Monazite, bastnäsite, and thorite mineral assemblages, with bastnäsite and thorite aggregates developed along monazite margins or within cavities; (k) Zircon, xenotime, and thorite mineral assemblages, with zircon fragmentation and secondary thorite aggregates developed along zircon margins or within cavities; (l) Epidote-altered feldspar with allanite, showing partial dissolution of allanite. (abbreviations: Zrt—zircon; Fer—ferrocolumbite; Bas, Bst—bastnäsite; Aln—allanite; Mon, Mnz—monazite; Ap—apatite; Xen—xenotime; Thr—thorite aggregates; Ttn—sphene; Ab—albite; Kfs—microcline; Mt—magnetite; Bt—biotite).

The bedrock of the weathering profile is a medium- to fine-grained monzonitic granite, characterized by a mineral assemblage dominated by plagioclase (45–50%), microcline (30–35%), and quartz (20–25%), with minor biotite and muscovite. Plagioclase occurs as subhedral tabular crystals with random orientation, exhibiting polysynthetic twinning and sericite alteration, with surfaces obscured by alteration products. Microcline is subhedral and tabular, also randomly oriented, and shows advanced saussuritization, resulting in weathered and altered surfaces. Quartz is anhedral, occurring as interstitial grains with undulose extinction and relatively fresh, unaltered surfaces. Biotite and muscovite are present as flaky crystals in a scattered distribution, partially replaced by chlorite and opaque minerals (Fig 3a). Feldspar minerals exhibit moderate alteration, including sericite and chlorite replacement. Accessory minerals include apatite, zircon, epidote, magnetite, sphene, and monazite (Fig 3b–3h).

The weathering profile hosts key light rare earth element (LREE)-enriched minerals including bastnäsite, apatite, allanite, monazite, sphene, and thorite, while zircon, xenotime, and ferrocolumbite represent the primary heavy rare earth element (HREE)-enriched phases (Fig 3). In the bedrock, monazite occurs as anhedral grains (10–60 μm) (Fig 3b). Zircon exhibits euhedral to subhedral habits (20–80 μm) (Fig 3c, 3d, 3g, 3h). Bastnäsite and ferrocolumbite are anhedral, either growing along the margins of other minerals or filling cavities as inclusions (10–50 μm) (Fig 3c, 3d, 3g). Apatite is anhedral to subhedral, with some grains included within zircon (10–50 μm) (Fig 3d, 3g, 3h). Sphene is anhedral (20–30 μm) (Fig 3d), while allanite is anhedral and developed along monazite margins or within microcline alteration fractures (Fig 3b, 3e). Accessory thorite occurs as secondary minerals filling interstitial spaces (Fig 3f). In the semi-weathered zone, sphene, bastnäsite, and ferrocolumbite are absent. Instead, abundant zircon and monazite (euhedral to subhedral, 10–80 μm) are observed, with zircon showing dissolution and reprecipitation textures (Fig 3i). Amorphous thorite aggregates and bastnäsite fill mineral margins or cavities (Fig 3j, 3k). Allanite, hosted within epidotized feldspar, exhibits partial dissolution (Fig 3l). These observations highlight depth-dependent mineralogical transformations across the weathering profile.

### Major elements

The major element composition of the weathering profile is presented in Table 1. The rock samples exhibit a wide range of silica (SiO₂) concentrations, from 62.23 to 78.34 wt%, with potassium oxide (K₂O) ranging from 3.99% to 6.02 wt%, sodium oxide (Na₂O) from 0.123% to 3.795 wt%, iron oxide (Fe₂O₃) from 0.472% to 5.12 wt%, calcium oxide (CaO) from 0.044% to 0.468 wt%, and magnesium oxide (MgO) from 0.043% to 0.248 wt%. To quantify weathering intensity, the Chemical Index of Alteration (CIA) [47] was calculated using the following equation:


$$CIA\;=\;100\;\times \;[Al_{2}O_{3}/(Al_{2}O_{3}\;+\;CaO\;+\;Na_{2}O\;+\;K_{2}O)]$$ (2)

**Fig 4:**
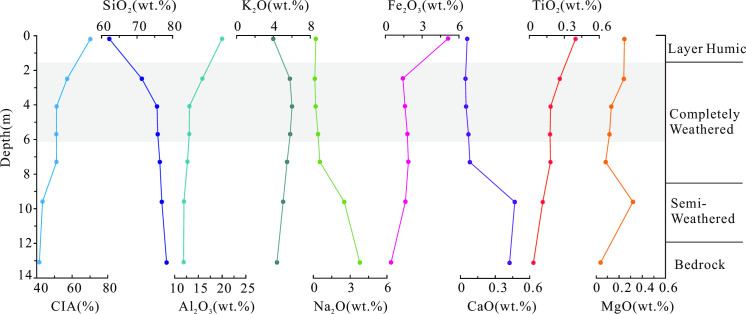
Major Element Composition Across the Weathering Profile.

CIA values increase progressively from the bedrock to the surface soil, indicating intensifying weathering. Notably, SiO₂ concentrations decrease from 78.34 wt% in the bedrock to 62.23 wt% in the surface soil, while Al₂O₃ increases from 11.9% in the bedrock to 20% in the surface soil (Fig 4). In the weathering profile, the depletion of Na₂O and CaO is particularly pronounced due to the weathering of feldspar and other silicate minerals. K₂O exhibits a unique trend, initially increasing to a maximum of 6.02 wt% in the middle of the fully weathered zone before decreasing to 3.99 wt% in the surface soil. In contrast, Al₂O₃, TiO₂, and Fe₂O₃ behave as immobile components, showing systematic increases from the bedrock to the surface soil. These trends reflect the progressive breakdown of silicate minerals, particularly feldspars, and the leaching of alkali and alkaline-earth elements as they form soluble compounds. The relative enrichment of immobile components, particularly in the upper weathering profile, underscores the intensifying weathering process and the preferential loss of mobile elements.

### REE contents

**Fig 5:**
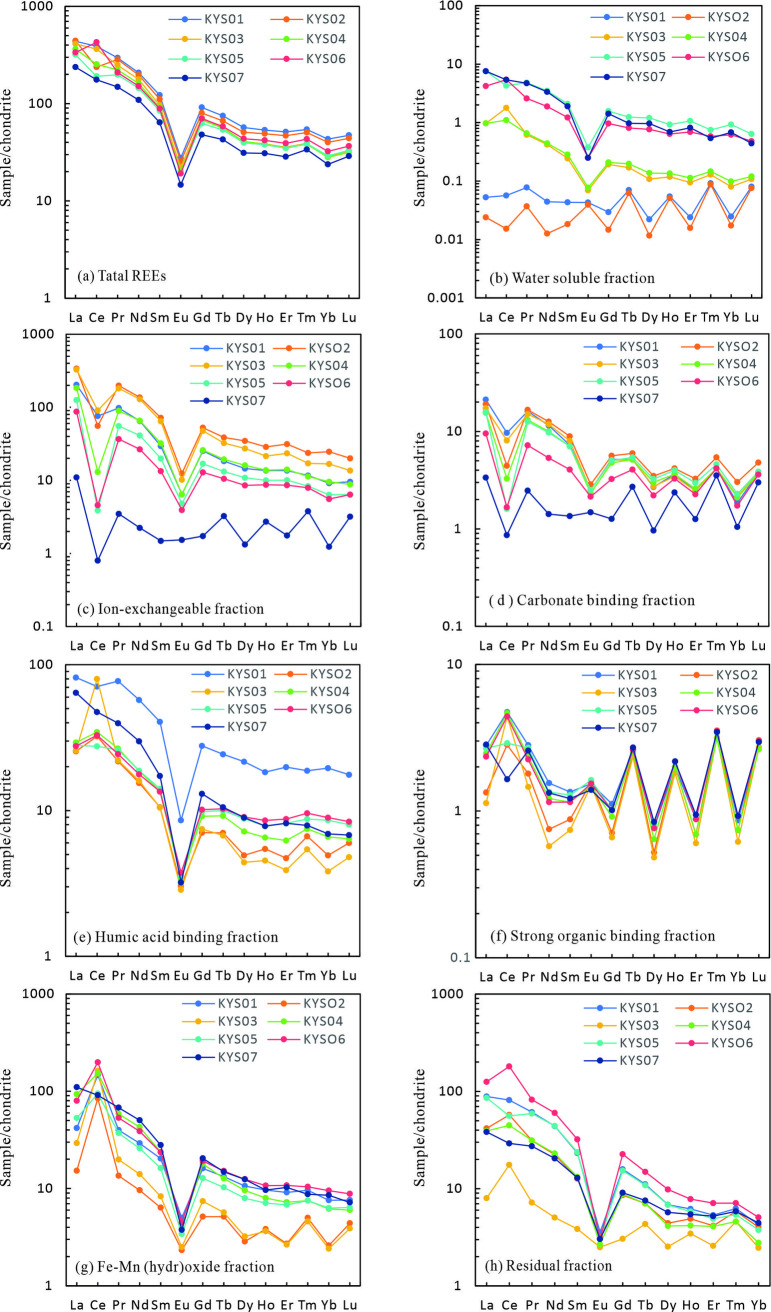
Chondrite-Normalized Rare Earth Element Distribution Across the Weathering Profile.

The bedrock of the weathering profile contains a total rare earth element (REE) concentration of 316.79 mg/kg (Table 2), which is higher than the average value of 229 mg/kg for South China granites [15], indicating a favorable foundation for REE mineralization. The chondrite-normalized REE pattern of the weathering profile shows a pronounced right-leaning trend (Fig 5a), characteristic of light rare earth element (LREE) enrichment. The REE composition in the weathering profile inherits the geochemical signature of the bedrock but exhibits systematic enrichment during weathering. All samples from the weathering profile show higher REE concentrations than the bedrock, with the semi-weathered zone showing a significant increase to 561.38 mg/kg. The highest REE concentration (630.87 mg/kg) is observed in the surface soil layer, which is attributed to plant root uptake of REEs[47]. From the bedrock to the surface soil, LREEs are relatively enriched, as indicated by LREE/HREE ratios ranging from 2.54 to 3.48 and (La/Yb)_N_ values from 9.98 to 14.51. The semi-weathered zone shows a strong positive Ce anomaly (δCe = 1.61), while the fully weathered zone exhibits a slight negative Ce anomaly (δCe = 0.67–1.14, average 0.87). The bedrock and surface soil show no significant Ce anomaly. Notably, the entire profile exhibits a strong negative Eu anomaly (δEu = 0.24–0.27) (Table 2).

### REE speciation

**Fig 6:**
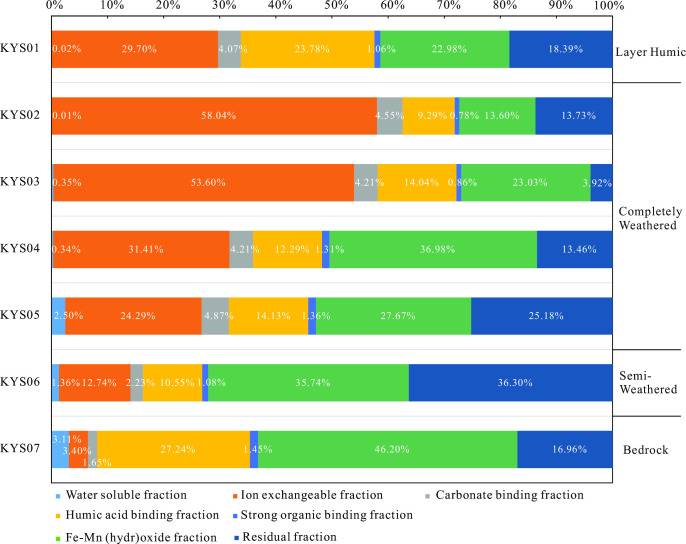
Cumulative Percentage of Different Rare Earth Element Fractions Across the Weathering Profile.

In the weathering profile, rare earth elements (REEs) predominantly occur in ion-exchangeable, residual, iron-manganese oxide-bound, and humic acid-bound forms, while water-soluble, carbonate-bound, and strongly organic-bound REEs are relatively scarce (Fig 6). Chondrite-normalized REE patterns for different REE fractions further highlight distinct geochemical behaviors among these fractions.

Ion-exchangeable rare earth elements (REEs) are those that can be exchanged with other cations in electrolyte solutions and are readily adsorbed onto clay minerals [48,49], making them the primary REE occurrence in ion-adsorption type REE deposits. In the studied weathering profile, the ion-exchangeable REE content increases from the bedrock to the fully weathered zone, peaking at 58.04% of the total REEs in the upper part of the fully weathered layer, before dropping to 29.7% in the surface soil layer (Fig 6). The chondrite-normalized REE pattern for the ion-exchangeable fraction shows a right-leaning trend with a notable negative Ce anomaly (Fig 5c). From the bedrock to the surface soil, the LREE/HREE and (La/Yb)_N_ values of the ion-exchangeable REEs increase progressively (Table 3). These findings highlight the significant role of ion-exchangeable REEs in the weathering profile and their potential for enrichment and differentiation during weathering processes.

**Fig 7:**
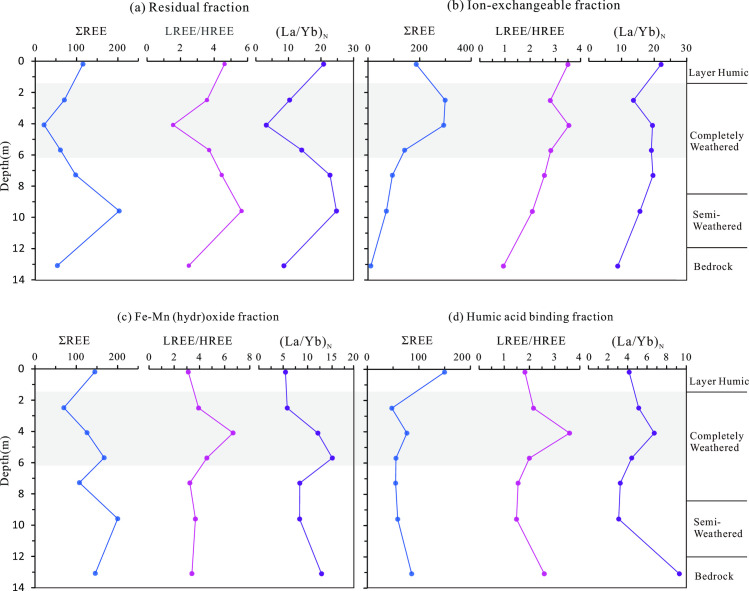
Variation of ΣREE, LREE/HREE, and (La/Yb)_N_ Ratios with Depth Across the Weathering Profile.

Iron-manganese oxide-bound rare earth elements (REEs) refer to those associated with iron and manganese oxides through adsorption or precipitation. As shown in Fig 6, this fraction constitutes a significant REE reservoir in the weathering profile, accounting for 46.2% of the total REEs in the bedrock. The proportion of iron-manganese oxide-bound REEs decreases progressively from the bedrock to the surface soil layer (Fig 6). The chondrite-normalized REE pattern for iron-manganese oxide-bound REEs exhibits a right-leaning trend, indicative of light rare earth element (LREE) enrichment (Fig 5g). The LREE/HREE ratios for iron-manganese oxide-bound REEs in the weathering profile range from 3.11 to 6.68 (average 4.21), slightly higher than the whole-rock LREE/HREE ratio of 2.54 in the bedrock. The (La/Yb)_N_ values for iron-manganese oxide-bound REEs in the weathering profile span from 5.49 to 15.09 (average 9.23), aligning with the whole-rock (La/Yb)_N_ value of 9.98 in the bedrock. However, these values show a gradual decrease with increasing weathering intensity (Table 3, Fig 7). A notable positive Ce anomaly (δCe values between 2.07 and 6.71) characterizes the iron-manganese oxide-bound REEs from the semi-weathered zone to the surface soil layer. This anomaly provides insights into redox conditions and the selective enrichment of certain REEs during weathering.

Humic acid-bound rare earth elements (REEs) refer to those complexed with humic acids. In the weathering profile, the proportion of humic acid-bound REEs varies significantly. It accounts for 27.24% of the total REEs in the bedrock, decreases to 9.29–14.13% in the semi-weathered to fully weathered zones, and increases again to 23.78% in the surface soil layer (Fig 6). The chondrite-normalized REE pattern for humic acid-bound REEs shows a right-leaning trend, indicating LREE enrichment. The LREE/HREE ratios range from 1.49 to 3.60, and the (La/Yb)_N_ values span from 3.10 to 6.74, which are lower than the whole-rock (La/Yb)_N_ value of 9.31 in the bedrock (Table 3, Fig 5e). A positive Ce anomaly is observed in the humic acid-bound REEs, with δCe values ranging from 0.89 to 3.35 (average 1.44). These geochemical characteristics highlight the dynamic behavior of REEs during weathering and their interaction with organic matter.

Strongly organic-bound rare earth elements (REEs) are those complexed with organic matter. They show light rare earth element (LREE) enrichment with significant variability in elemental concentrations (Table 3, Fig 5f), yet contribute less than 2% to the weathering profile (Fig 6).

Carbonate-bound REEs, associated with carbonate minerals, also exhibit LREE enrichment but with a notable negative Ce anomaly (Table 3, Fig 5d). Their proportion in the weathering profile increases from 1.65% in the bedrock to 4.55% in the surface soil, yet remains below 5% (Table 3, Fig 6).

Water-soluble REEs exist as free ions and show LREE enrichment (Fig 5b). However, their concentrations are minimal throughout the profile, contributing less than 5% to the total REE budget (Fig 6).

The residual rare earth elements (REEs) in the weathering profile represent those that remain undecomposed or newly formed during weathering, stably residing in rare-earth-bearing and rock-forming minerals. In the studied profile, the residual REE content exhibits significant variation: it constitutes 16.96% of the total REEs in the bedrock, increases to 36.3% in the semi-weathered zone, decreases to 3.92% in the fully weathered zone, and rises again to 13.73% in the surface soil layer (Fig 6). The chondrite-normalized REE pattern for residual REEs shows a right-leaning trend, indicative of LREE enrichment. The LREE/HREE ratios range from 1.56 to 5.64, and the (La/Yb)_N_ values span from 3.23 to 24.80 (Table 3, Fig 5h), further confirming the preferential enrichment of LREEs in the residual fraction.

## Discussion

### The spatial variability and migration–Immobilization mechanisms of rare earth elements in the weathering profile

The modes of occurrence of rare earth elements in ionic adsorption deposits show pronounced spatial variability [8,50]. The ion exchange, iron-manganese oxide, and humic acid binding forms collectively form the system through which rare earth elements migrate and become immobilized in geological processes [51–53]. The proportions of different forms of rare earth elements in the Kuanyu rare earth deposit closely resemble those in well-known ionic adsorption-type deposits in southern China, including Renju in Guangdong [25], Chongzuo in Guangxi [54], Nanningdu in Jiangxi [39], and southern Yunnan [52]. In all these deposits, the rare earth elements are predominantly in the ion exchange state, followed by the iron-manganese oxide binding state, and finally the humic acid binding state. The vertical distribution of elements in the soil shows that the organic-rich layer at the top of the weathered crust (0-0.5m) is influenced by biogeochemical processes. Substances released by plant roots, such as citric and oxalic acids, work together with humic acids to significantly reduce the proportion of rare earth elements in the ion exchange state to 28–32% through a process of forming complexes and competing for adsorption sites. Additionally, this process activates surface sites on iron-manganese oxides, causing the proportion of rare earth elements bound to these oxides to increase to 35–40% [3,52,55–58]. The completely weathered layer (0-3m) is characterized by the extensive development of layered silicate clay minerals such as kaolinite and montmorillonite (>35%), which create a strong ion exchange capacity. In this layer, the proportion of rare earth elements that can be exchanged ions can reach 55–62% of the total rare earth content. This high ion exchange capacity is due to the abundant negative charge sites on the surfaces of these clay minerals and the ability of their interlayers to accommodate exchangeable cations [50,59,60]. With increasing depth into the transitional weathering zone (3-8m), the decrease in oxidation-reduction potential (Eh) from +500mV to +200mV promotes the lattice restructuring of iron-manganese oxides (such as goethite and pyrolusite), leading to an increase in the proportion of rare earth elements bound to these oxides to 38–42%. Additionally, the residual fraction (RES) rises from 8% in the completely weathered layer to 22% in the transitional zone, indicating the conversion of rare earth elements into more stable mineral phases [52,61].

Furthermore, in warm and humid climates, despite the potential for heavy rainfall to induce rapid migration of rare earth elements (REEs), the surface humic layer of the weathering crust effectively retards the leaching of REEs through the establishment of an “organic-mineral composite immobilization system” [3,52,62]. For instance, in the weathering profile of dolomite in Guangxi, the total rare earth content in the surface soil (0–20 cm) is 1–2 orders of magnitude higher than that in the bedrock, with an average of 149.834 ppm compared to 2.11 ppm [62]. The REE content in the humic layer (0-1.5m) of the Kuanyu deposit is 1.9 times that of the bedrock, demonstrating similar characteristics, which aligns with the findings of on the redistribution of REEs in humic layers [52]. This is primarily attributed to the triple fixation mechanism of the humic acid-clay mineral composite system [50]. The high precipitation (>1500 mm/yr) and permeability of the subtropical climate facilitate the rapid decomposition of primary minerals (such as feldspar and apatite), releasing REEs. Simultaneously, the continuous input of humic acids and the highly developed clay minerals establish a “release-fixation” dynamic equilibrium [3].

### REE fractionation during dissolution of primary REE-bearing minerals

The fractionation of rare earth elements (REEs) during granite weathering is closely related to the stability of primary REE-bearing minerals and the formation of secondary minerals [40,63,64] From the semi-weathered zone to the fully weathered zone, there is a rapid decrease in the residual REE content, indicating significant weathering and dissolution of easily alterable minerals (Fig 3e, 3l). These minerals, such as sphene, bastnäsite, and allanite, are relatively enriched in light rare earth elements (LREEs). The released LREEs migrate through vertical leaching, leading to a decrease in the LREE/HREE and (La/Yb)_N_ ratios of the residual REEs from the semi-weathered to the fully weathered zones, thereby promoting LREE-HREE fractionation. In the fully weathered zone to the surface humus layer, weathering is complete, and even resistant minerals rich in heavy rare earth elements (HREEs) continue to dissolve. Influenced by plant roots and humic acids, the total residual REE content, along with LREE/HREE and (La/Yb)_N_ ratios, increase again. Fig 6 shows a significant increase in residual REE content from the bedrock to the semi-weathered layer, indicating the formation of secondary REE minerals during this stage. Table 3 shows that in the semi-weathered layer, the content of LREEs (except Eu) increases markedly, especially for Ce, whose content exceeds the bedrock level by more than six times. Backscattered electron images reveal partial dissolution of easily weathered, LREE-rich minerals in the semi-weathered layer, with secondary bastnäsite and thorium minerals filling dissolution seams and margins of zircon and monazite. These secondary thorium minerals are poorly crystallized (Fig 3i-3k), resembling those reported by Du et al. in weathered profiles of metamorphic granites in Western Australia [65]. It is inferred that thorium, derived from the breakdown of bastnäsite, apatite, monazite, and zircon, combines with LREEs released from the early-stage dissolution of light REE minerals and LREEs leached from the upper profile to form thorium crystals. Due to thorium’s low mobility [65], these crystals mainly occur at the edges or in dissolution seams of zircon, monazite, and apatite (Fig 3f, 3i–3k). The formation of these LREE-rich secondary thorium crystals and bastnäsite likely explains the increased total residual REE content, positive Ce anomalies, and elevated LREE/HREE and (La/Yb)_N_ ratios in the semi-weathered layer. Thus, the weathering of primary REE minerals and the formation of secondary minerals are key drivers of REE fractionation in the weathering profile. From the bedrock to the semi-weathered layer, the LREE/HREE and (La/Yb)_N_ values of iron-manganese oxide-bound and humic acid-bound REEs decrease, indicating HREE enrichment. The leached HREEs are predominantly retained by iron-manganese oxides and humic acids.

### REE fractionation in mineralisation

Ion-exchangeable rare earth elements (REEs), the most economically viable REE fraction [66,67], show a remarkable increase in content from the bedrock to the fully weathered layer in the weathering profile, reaching up to 58% of the total REEs in the upper part of the fully weathered layer (Fig 6). The LREE/HREE and (La/Yb)_N_ ratios of ion-exchangeable REEs exhibit an increasing trend from the bedrock to the surface soil layer, indicating a preferential enrichment of LREEs in this fraction and highlighting its LREE-enriched nature (Fig 7b). Specifically, the LREE/HREE ratios of ion-exchangeable REEs range from 2.09 to 3.54 (average 2.88), aligning with the whole-rock LREE/HREE ratio of 2.54 in the bedrock. This suggests that the REE composition of the ion-exchangeable fraction inherits the geochemical signature of the parent rock. However, the (La/Yb)_N_ values of ion-exchangeable REEs in the weathering profile, varying from 13.72 to 22.20 (average 18.37), are significantly higher than the whole-rock (La/Yb)_N_ value of 9.98 in the bedrock. This disparity implies that ion-exchangeable REEs promote LREE enrichment. Consequently, as weathering and leaching processes intensify, ion-exchangeable REEs drive the fractionation of LREEs and HREEs [52,68,69].

Iron-manganese oxide-bound rare earth elements (REEs) are a significant form of REE occurrence in ion-adsorption type REE deposits. In the weathering profile, the content of iron-manganese oxide-bound REEs is as high as 46.20% in the bedrock layer and remains substantial at an average of 24.75% in the fully weathered layer, second only to ion-exchangeable REEs (Fig 6). This highlights the importance of iron-manganese oxides as a major REE reservoir in these deposits. The iron-manganese oxide-bound REEs exhibit a light rare earth element (LREE)-enriched pattern [25,52]. From the bedrock to the surface soil layer, the LREE/HREE and (La/Yb)_N_ ratios of this fraction show a decreasing trend (Fig 7c). In the early stages of weathering, easily weathered minerals such as bastnäsite, sphene, and allanite decompose preferentially [25]. Since these minerals are LREE-rich, the released LREEs are immobilized by iron-manganese oxides, leading to LREE enrichment in the iron-manganese oxide fraction. As weathering progresses, heavy rare earth elements (HREEs) are progressively leached, causing the (La/Yb)_N_ ratios to decrease [52]. From the fully weathered layer to the surface soil layer, the content of iron-manganese oxide-bound REEs increases, while their LREE/HREE and (La/Yb)_N_ ratios decrease, indicating a trend of HREE enrichment. This suggests that iron-manganese oxides play a key role in the fractionation and redistribution of REEs during weathering.

In the weathering profile, ion-exchangeable REEs show an increasing trend in LREE/HREE and (La/Yb)_N_ ratios from the bedrock to the surface soil layer, indicating a preferential enrichment of LREEs in this fraction. Conversely, iron-manganese oxide-bound REEs exhibit a decreasing trend in (La/Yb)_N_ ratios across the profile, suggesting a tendency to enrich HREEs. However, in the middle of the fully weathered layer, the (La/Yb)_N_ ratio of iron-manganese oxide-bound REEs increases abruptly before gradually decreasing again. This is because in the lower part of the weathering profile, REE minerals decompose less, and iron-manganese oxides mainly retain HREEs leached from the upper layers, while LREEs are predominantly present in residual and ion-exchangeable forms [52,70]. As weathering progresses into the upper part of the fully weathered layer, rapid dissolution of residual REEs releases LREEs, which quickly bind to iron-manganese oxides, causing the (La/Yb)_N_ ratio to surge [70]. Subsequently, as the content of ion-exchangeable REEs increases rapidly and LREEs become enriched in this fraction, the content of iron-manganese oxide-bound REEs decreases, along with a reduction in LREE/HREE and (La/Yb)_N_ ratios, indicating a shift towards HREE enrichment [71]. This also suggests that the decomposition of REE minerals and the formation of clay minerals do not occur synchronously. Iron-manganese oxides exhibit a strong positive Ce anomaly (δCe = 2.07–6.71), indicating that Ce is easily oxidized and bound to iron-manganese oxides. Studies suggest that iron-manganese oxides adsorb REEs through inner-sphere complexation. Compared to LREEs, HREEs have smaller ionic radii and stronger hydrolysis abilities, giving them a higher affinity for iron oxides [72]. Despite the influence of the parent rock’s REE composition, iron-manganese oxide-bound REEs still show LREE enrichment. The pronounced positive Ce anomaly in the iron-manganese oxide-bound fraction and the negative Ce anomaly in the ion-exchangeable fraction indicate that Ce differentiation is related to iron-manganese oxides. Previous studies have proposed that iron-manganese oxides can oxidize Ce³⁺ to Ce⁴ ⁺ , forming CeO₂ and separating it from other REE³⁺ [61–62]. It forms soluble complexes and is preferentially adsorbed by iron and manganese hydroxides, leading to the relative enrichment of Ce in iron and manganese oxides. As a result, the Ce/La ratio in iron and manganese oxides is significantly higher than the Ce/La ratio of the total rare earth elements [71,73]. This highlights that iron-manganese oxides play a significant role in the enrichment and differentiation of LREEs and HREEs in the weathering profile and also contribute to Ce differentiation, which is related to the content of iron-manganese oxides [74].

In the weathering profile, the humic acid-bound rare earth elements (REEs) account for a significant proportion of the total REEs, reaching as high as 12.36% in the fully weathered layer. From the semi-weathered layer to the surface layer, the LREE/HREE ratios of humic acid-bound REEs (1.49–3.60, average 2.11) are comparable to the whole-rock LREE/HREE ratio of 2.54 in the bedrock, while the (La/Yb)_N_ ratios (3.10–6.74) are significantly lower than the whole-rock (La/Yb)_N_ ratio of 9.98 in the bedrock. This indicates that humic acid-bound REEs tend to enrich heavy rare earth elements (HREEs). The rapid increase in humic acid-bound REE content in the surface soil layer, coupled with a decrease in LREE/HREE and (La/Yb)_N_ values, further corroborates the tendency of humic acid to enrich HREEs. This enrichment is attributed to the fact that as the ionic radius of rare earth elements decreases from La to Lu, the binding between rare earth elements and organic matter becomes more stable, with HREEs having a stronger affinity for organic matter than LREEs [75]. Humic acid – bound rare earth elements also show a certain degree of Ce positive anomaly. This is because Ce is preferentially adsorbed by iron oxides related to humic substances in an oxidative environment. In contrast, other rare earth elements are leached out, which leads to an increase in the Ce/La ratio in humic substances and results in a Ce positive anomaly [71,73]. However, similar to iron-manganese oxide-bound REEs, the humic acid-bound REEs still show an enrichment of LREEs due to the influence of the parent rock’s REE composition. The pronounced positive Ce anomaly in the iron-manganese oxide-bound fraction and the negative Ce anomaly in the ion-exchangeable fraction indicate that Ce differentiation is associated with iron-manganese oxides.

The weathering profile inherits the chondrite-normalized REE pattern of the parent rock, showing a right-leaning distribution with LREE enrichment. However, distinct differences in REE distribution patterns are observed among different REE fractions (Fig 6). Ion-exchangeable REEs show a clear LREE-enriched trend, with LREE/HREE and (La/Yb)_N_ values increasing progressively from the bedrock to the surface soil layer. Iron-manganese oxide-bound and humic acid-bound REEs exhibit HREE-enriched tendencies, with lower LREE/HREE and (La/Yb)_N_ values compared to the parent rock. Despite these differences, all three REE fractions show LREE enrichment overall, due to the geochemical signature inherited from the parent rock.

### Genetic model of the Kuanyu Ion-adsorption rare earth deposit

**Fig 8:**
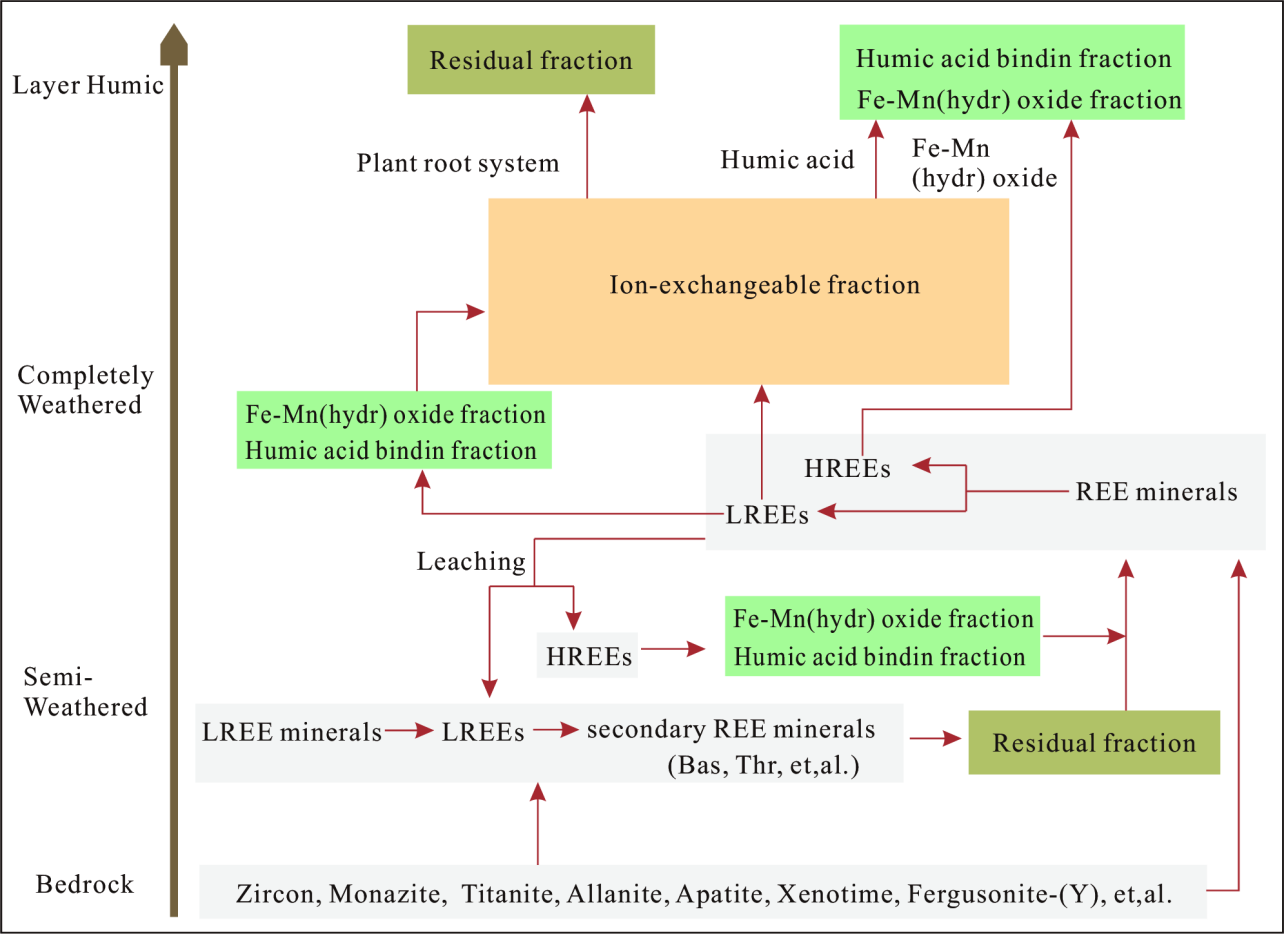
Genetic Model of the Kuanyu Ion-Adsorption Rare Earth Deposit.

The Kuanyu ion-adsorption rare earth deposit exhibits a unique genetic model (Fig 8). As weathering progresses, various REE-bearing minerals in the bedrock are continuously decomposed. In the semi-weathered zone, minerals rich in light rare earth elements (LREEs), such as apatite, sphene, and bassinet, begin to break down. Some of the released REEs form insoluble secondary REE minerals. Additionally, LREEs leached from the upper layers also contribute to the formation of secondary REE minerals in this zone, including secondary bassinet and thorite aggregates. These processes lead to a significant increase in the residual REE content, with elevated LREE/HREE and (La/Yb)_N_ ratios, indicating LREE enrichment. In contrast, heavy rare earth elements (HREEs) leached from the upper layers primarily bind to iron-manganese oxides and humic acids, resulting in lower LREE/HREE and (La/Yb)_N_ ratios for these fractions. In the fully weathered layer, most primary REE minerals are completely decomposed, leading to a substantial reduction in residual REE content. Due to the asynchronous decomposition of REE minerals and rock-forming minerals, a large amount of LREEs initially bind to iron-manganese oxides and humic acids. Subsequently, with the breakdown of rock-forming minerals and the formation of clay minerals, ion-adsorption REEs increase rapidly and show a preference for LREE enrichment. The content of iron-manganese oxide-bound and humic acid-bound REEs decreases, with a gradual trend towards HREE enrichment. In the surface soil layer, influenced by plant roots, iron-manganese oxides, and humic acids, the content of residual, iron-manganese oxide-bound, and humic acid-bound REEs increases, forming a surface-enriched layer of REEs. Here, residual REEs are LREE-enriched, while iron-manganese oxide-bound and humic acid-bound REEs show HREE-enriched tendencies.

## Conclusions

This study offers preliminary insights into the fractionation mechanisms of rare earth elements (REEs) within the Kuanyu weathering profile, yet acknowledges limitations due to the restricted number of sampling points, suggesting that further investigation is needed to better understand REE migration and enrichment processes.

1. The Kuanyu ion-adsorption REE deposit is characterized as an LREE-type. REE concentrations progressively increase from the bedrock to the surface soil layer, with the fully weathered layer serving as the primary zone of REE enrichment.
2. The weathering of primary REE-bearing minerals and the formation of secondary minerals within the weathering profile are crucial drivers of REE fractionation.
3. Within the Kuanyu weathering profile, ion-exchangeable REEs show a preference for LREE enrichment, whereas iron-manganese oxide-bound and humic acid-bound REEs exhibit a tendency to enrich HREEs. However, all three fractions display an overall LREE enrichment pattern, influenced by the REE composition of the parent rock. Clay minerals, iron-manganese oxides, and humic acids are the dominant factors affecting REE fractionation during the mineralization process of the Kuanyu ion-adsorption REE deposit.
